# *CYP2C19* Gene Profiling as a Tool for Personalized Stress Ulcer Prophylaxis With Proton Pump Inhibitors in Critically Ill Patients - Recommendations Proposal

**DOI:** 10.3389/fmed.2022.854280

**Published:** 2022-07-11

**Authors:** Petra Bořilová Linhartová, Ondřej Zendulka, Jaroslav Janošek, Natálie Mlčůchová, Michaela Cvanová, Zdeněk Daněk, Radek Kroupa, Ladislava Bartošová, Břetislav Lipový

**Affiliations:** ^1^RECETOX, Faculty of Science, Masaryk University, Brno, Czechia; ^2^Clinic of Maxillofacial Surgery, Faculty of Medicine, Institution Shared With University Hospital Brno, Masaryk University, Brno, Czechia; ^3^Department of Pharmacology, Faculty of Medicine, Masaryk University, Brno, Czechia; ^4^Faculty of Medicine, Center for Health Research, University of Ostrava, Ostrava, Czechia; ^5^Faculty of Medicine, Institute of Biostatistics and Analyses, Masaryk University, Brno, Czechia; ^6^Department of Internal Medicine and Gastroenterology, Faculty of Medicine, Institution Shared With University Hospital Brno, Masaryk University, Brno, Czechia; ^7^Department of Burns and Plastic Surgery, Faculty of Medicine, Institution Shared With University Hospital BrnoMasaryk University, Brno, Czechia

**Keywords:** critical care, personalized therapy, stress ulcer prophylaxis, proton pump inhibitors, pharmacogenetics, gene polymorphism, poor metabolizer, ultra-rapid metabolizer

## Abstract

To this date, there are no recommendations for personalized stress ulcer prophylaxis (SUP) in critical care that would take the patient's individual genetic predispositions into account. Of drugs used for this purpose, proton pump inhibitors (PPIs) are the first-choice drugs in intensive care unit patients. The degradation of proton pump inhibitors is mediated by cytochrome P450 (CYP) enzymes; in particular, CYP2C19 and, to a lesser extent, CYP3A4 are involved. Expression and metabolic activity of, namely in, CYP2C19 is significantly affected by single nucleotide polymorphisms, the drug metabolization rate varies greatly from ultrarapid to poor and likely influences the optimal dosage. As these CYP2C19 predictive phenotypes via *CYP2C19* haplogenotypes (rs12248560/rs4244285) can be relatively easily determined using the current standard equipment of hospital laboratories, we prepared a set of recommendations for personalized PPI-based stress ulcer prophylaxis taking into account the patient's CYP2C19 predictive phenotype determined in this way. These recommendations are valid, in particular, for European, American and African populations, because these populations have the high representations of the *CYP*2*C*19^*^17 allele associated with the overexpression of the *CYP2C19* gene and ultrarapid degradation of PPIs. We propose the *CYP2C19* gene profiling as a tool for personalized SUP with PPI in critically ill patients.

## Introduction

Stress ulcers are a relatively common complication in intensive care units (ICU) patients – up to 90% of such patients develop some degree of gastric mucosal damage within as few as 3 days at ICU (1–3). Most such erosions are only superficial and asymptomatic; however, a significant percentage of patients (ranging from 0.6 to 7.0%) develop clinically relevant gastrointestinal bleeding (GIB) (3–8). The risk factors for GIB development in critically ill patients include the length of ICU stay, elevated creatinine on ICU admission (9), mechanical ventilation >48 h, coagulopathy, acute kidney injury, chronic renal failure, acute hepatic failure, hypotension, history of alcohol abuse, and prolonged nasogastric tube placement (10). GIB is associated with adverse outcomes and is an important indicator of morbidity and mortality in these patients (4, 11, 12). Patients with the above risk factors may benefit from increased vigilance for the development of GIB; stress ulcer prophylaxis (SUP) is generally recommended in such patients and is widely used in ICU (13–15). Recently, a clinical practice guideline for GIB prophylaxis in critically ill patients was published (16).

Proton pump inhibitors (PPIs) approved by the Food and Drug Administration [i.e., omeprazole, esomeprazole, lansoprazole, pantoprazole, and rabeprazole; (17)] are reasonable choices for SUP in ICU patients. PPIs are metabolized (degraded) in the liver by cytochrome P450 (CYP) enzymes, the activity of which is influenced by both external and internal factors. This biotransformation to inactive metabolites is primarily mediated by the isoenzyme CYP2C19 and, to a lesser extent, by CYP3A4. Rabeprazole, being predominantly biotransformed by non-enzymatic metabolic pathways, is an exception to this rule (18, 19). The genetically conditioned activity of these enzymes (and, in effect, the PPI degradation rate) is, therefore, likely to play an important role in the effectiveness of SUP in a particular patient. For this reason, the metabolic activity of the CYP enzymes should be taken into account in patient management. This is in line with the concept of personalized medicine, which is currently considered the best way to the improvement of treatment effectiveness in general.

To this date, however, **no recommendations taking into account the patient's individual predispositions for personalized SUP in critical care are available**. In this paper, we aim (i) to assemble the available data on personalized PPI therapy taking into account *CYP2C19* gene variability, (ii) to propose a concept for the personalized SUP by PPIs in ICU patients according their *CYP2C19* gene profile, and (iii) to estimate the distribution of the *CYP2C19* haplogenotype frequencies affecting PPI metabolization in various populations and, thus, to determine populations that would most benefit from such an approach. In this way, this paper aims to contribute to the implementation of the principles of personalized medicine into clinical practice.

## Methods

The proposed concept for the personalized SUP in ICU patients is based on the data from the “Clinical Pharmacogenetics Implementation Consortium (CPIC) Guideline for CYP2C19 and Proton Pump Inhibitor Dosing” (20), “Proton Pump Inhibitors: U.S. Food and Drug Administration-Approved Indications and Dosages for Use in Adults” (17, 21), information from PharmGKB database and from The Dutch Pharmacogenetics Working Group (DPWG) (22), and recent scientific studies (23–28).

The *CYP2C19* haplogenotypes from diplotype frequencies estimated using the equation describing Hardy-Weinberg equilibrium based on reported allele frequencies from the PharmGKB database in nine populations worldwide (22); were calculated. Our previous study in the Czech (Central European) population (29) and the largest study with experimentally determined *CYP2C19* haplogenotypes (30) were included for comparison. The frequencies of the extremely rare haplogenotypes with unknown phenotypes detected by Ionova et al. (30) were neglected for the purposes of this calculation. Differences in frequencies between populations were tested by the Chi-square exact test and Cramer's phi coefficient (ϕ) was used for measuring the effect size (the scale used for interpretation was as follows: 0.3 ≤ ϕ <0.5, medium effect; ϕ ≥ 0.5, large effect). The European population from the PharmGKB database was chosen as the reference population.

## Individual Predispositions to Ppi Metabolization Based on *Cyp2C19* Gene Variability

The interindividual variability of pharmacokinetic parameters leads to obvious differences between patients from the perspectives of the acidity-suppressing potential, the potential for drug interactions, and the clinical effectiveness of the drug. Alterations of both CYP2C19 and CYP3A4 activity can occur as a result of drug interactions or of the individual's genetic predispositions (31–33). The *CYP2C19* and *CYP3A4* gene expressions are significantly affected by genetic variability (most often, single nucleotide polymorphisms, SNPs). Even FDA highlights this on its website (namely, in the pages dedicated to drugs metabolized by this enzyme) as a caveat or even prescription limitation (34). In our opinion, however, the determination of only one SNP is not sufficient for an accurate definition of the patient's phenotype and for choosing the optimal SUP in line with principles of personalized medicine.

While *CYP3A4* polymorphisms with functional consequences are relatively rare in the population (≤5%) (35), some functional variants of the *CYP2C19* gene have a high prevalence across populations worldwide. So far, 11 variant alleles in the gene encoding this enzyme with clinical impact have been identified. Of these, only one, referred to as *CYP*2*C*19^*^17 (in the promotor region; NM_000769.2:c.-806C>T; rs12248560), was associated with the overexpression of the *CYP2C19* gene. Except for the allele ^*^17 and the standard “wild type” allele ^*^1, all other known variant *CYP2C19* alleles (^*^2, ^*^3, ^*^4, ^*^5, ^*^6, ^*^7, ^*^8, ^*^10, ^*^12, ^*^27) are associated with a reduced CYP2C19 enzymatic activity or its complete loss. Of these, the allele *CYP*2*C*19^*^2 (NM_000769.2:c.681G>A; p.Pro227Pro; rs4244285) is the most common one (35); in most populations, the representation of all remaining alleles of this group is negligible. The *CYP*2*C*19^*^2 point mutation in exon 5 leads to the formation of a new aberrant splice site in the amino acid sequence of the gene and, in effect, to the expression of a truncated and dysfunctional CYP2C19 protein (36).

It follows from the above that by determining the combination of these two SNPs (rs12248560/rs4244285), it is possible to predict the patient's ability to metabolize PPIs. However, to maximize the prediction accuracy, it is highly beneficial to determine not only haplotypes (combinations of alleles inherited from a single parent, i.e., present on the same DNA strand) but also haplogenotypes (i.e., combinations of selected genotypes, which can be associated with a specific predictive phenotype), see Supplementary Table 1 for a summary of six CYP2C19 predictive phenotypes based on two *CYP2C19* polymorphisms.

The haplogenotypes containing the *CYP*2*C*19^*^17 allele (i.e., ^*^17^*^17/^*^1^*^1 and ^*^1^*^17/^*^1^*^1) were associated with accelerated degradation of the active substances. They predict their carriers to be insensitive to the standard dosage of most PPIs, which may cause SUP failure; phenotypically, these individuals are so-called ultrarapid or rapid metabolizers (UM or RM). Homozygous carriers of two standard alleles (^*^1^*^1/^*^1^*^1) are referred to as extensive metabolizers (EM); those with one *CYP*2*C*19^*^2 allele (^*^1^*^1/^*^1^*^2) are referred to as intermediate metabolizers (IM) and clinically manifest through slower metabolism of the enzyme's substrates and, in effect, slower degradation of PPIs. The presence of two *CYP*2*C*19^*^2 alleles (^*^1^*^1/2^*^2) leads to the inactivity of the CYP2C19 enzyme – such carriers are referred to as poor metabolizers (PM); drugs degraded by CYP2C19 are not metabolized by carriers of this haplogenotype, which leads, besides its prolonged action, also to the accumulation of the drug in the organism and associated risk of unwanted side effects and drug-drug interactions.

When variant alleles with opposing effects on the CYP2C19 activity are present together (haplogenotype ^*^1^*^17/1^*^2), the phenotype is questionable; we have, therefore, called this phenotype AM [ambivalent metabolizer; (29)]. Limited data (from a study in patients using clopidogrel) suggest that the increased function allele *CYP*2*C*19^*^17 may not compensate for no function alleles such as *CYP*2*C*19^*^2 (37). This suggests that the AM individuals will likely metabolize PPIs at a slower pace than EM; however, the effect of PPI treatment in these patients needs to be further analyzed in more detail.

Haplogenotypes can be determined by polymerase chain reaction (PCR) based methods. These methods are nowadays, especially in association with the COVID-19 pandemic, widely available and if the method is introduced in the lab, determining haplogenotypes is a matter of hours with minimal costs. In addition, two genotyping platforms have been approved by FDA: the AmpliChip® CYP450 Test (Roche Molecular Systems, Inc., Pleasanton, CA) interrogating CYP2C19^*^2 and ^*^3 and the Infiniti® CYP2C19 Assay (AutoGenomics, Inc., Vista, CA) interrogating CYP2C19^*^2, ^*^3, and ^*^17.

## A Proposed Concept for the Personalized Sup By Ppi in Icu Patients According to Their *Cyp2C19* Gene Profile

While there are guidelines for personalized therapy with omeprazole, lansoprazole, and pantoprazole, the conclusions of the CPIC in 2020 contain no specific recommendations considering the patient's *CYP2C19* genetic profile for esomeprazole and rabeprazole (20). Generally, PPIs use in PM and IM patients results in higher systemic concentrations and possibly higher risk of adverse effects due to the decreased CYP2C19 enzyme activity (21). Based on the current literature, *CYP2C19* gene variability should be considered for all PPIs while, at the same time, it should be recognized that rabeprazole is least influenced by the *CYP2C19* variation (due to its different metabolization) (25). For this reason, we suggested rabeprazole to be the most reasonable choice for SUP in ICU patients with PM, IM, and AM predictive phenotypes. The summary of PPI dosage recommendations for SUP in ICU patients according to their *CYP2C19* gene profile is shown in Table 1, the full flowchart with complex recommendations is presented in Supplementary Figure 1.

**Table 1:** The summary of current proton pump inhibitors (PPIs) dosage recommendations for stress ulcer prophylaxis (SUP) in critically ill patients according their *CYP2C19* gene profile.

**PPI**	**Standard dose for SUP**	CYP2C19 predictive phenotypes^*^		
		**PM, IM, AM^#^**	**EM, RM**	**UM^‡^**
		***CYP2C19*** **enzyme with decreased function, poor degradation of some PPIs**	***CYP2C19*** **enzyme with normal/rapid function**	***CYP2C19*** **enzyme with increased function, ultrarapid degradation of some PPIs**
Omeprazole Lansoprazole Pantoprazole	20 mg/day (4–8 weeks) 30 mg/day (8 weeks) 20 mg/day (4–8 weeks)	See the footnote^†^	Initiate standard starting daily dose. Consider increasing dose by 50–100% for the treatment of *Helicobacter pylori* infection and erosive esophagitis. Daily dose may be given in split doses	Increase starting daily dose by 100%. Daily dose may be given in divided doses
Esomeprazole	20 mg/day (4–8weeks)	To this date, no specific recommendation considering the patient's *CYP2C19* genetic profile; however, esomeprazole is metabolized by *CYP2C19* enzyme		
Rabeprazole	20 mg/day (4–8weeks)	Standard dose. No specific recommendation considering the patient's CYP2C19 genetic profile, rabeprazole is metabolized predominantly non-enzymatically, which makes it suitable for all groups of metabolizers		

*PM, poor metabolizer; IM, intermediate metabolizer; AM, ambivalent metabolizer; EM, extensive metabolizer; RM, rapid metabolizer; UM, ultrarapid metabolizer; CYP, cytochrome P450*.

* Six CYP2C19 haplotypes/predictive phenotypes results from combination of two CYP2C19 polymorphisms (rs12248560 for detection of allele CYP2C19*17 / rs4244285 for detection of allele CYP2C19*2).

# The AM predictive phenotype for the*17*1/*1*2 CYP2C19 result haplogenotype is a provisional classification. The available evidence indicates that the CYP2C19*17 increased function allele is unable to completely compensate for the CYP2C19*2 no function allele.

‡ *If critically ill patient with UM phenotype is also H. pylori positive, standard dose of rabeprazole seems to be reasonable choice*.

† *The Clinical Pharmacogenetics Implementation Consortium guidelines recommend considering a 50% reduction in daily dose of first-generation PPIs to minimize the risk of adverse effects for chronic therapy (>12 weeks) in PMs, IMs and AMs (38). However, the data for determining the time point for reduction of the daily dose are insufficient. It follows that rabeprazole, which is dominantly degraded non-enzymatically, seems to be the most reasonable choice for SUP in critically ill patients with genetic predisposition to decreased function of the CYP2C19 enzyme*.

In addition, esomeprazole or rabeprazole showed better overall *Helicobacter pylori* eradication rates than first-generation PPIs (omeprazole, lansoprazole and pantoprazole) (39). Moreover, the eradication of *H. pylori* is facilitated in PMs compared to other metabolizers (28). A meta-analysis showed that a rabeprazole-based eradication program is less affected by the *CYP2C19* polymorphisms than the treatment by first-generation PPIs (27). Thus, increasing the dose of omeprazole, lansoprazole, or pantoprazole by 50–100% for the treatment of *H. pylori* infection in EMs and RMs is recommended. For *H. pylori* eradication therapy in UMs, the use of a 3-fold higher dose of omeprazole, a 4-fold higher dose of lansoprazole, 5-fold higher dose of pantoprazole, or dose increase by 50–100% in esomeprazole is recommended (20, 22). Hence, in critically ill *H. pylori* positive patients, the standard dose of rabeprazole seems to be a reasonable choice.

## Distribution of the *Cyp2C19* Haplogenotype Frequencies Affecting Ppi Metabolization in Various Populations

According to Lewis et al. (40), the *CYP*2*C*19^*^17 allele does not coexist in the haplotype together with the *CYP*2*C*19^*^2 allele – they are in a so-called link disequilibrium. It follows that it is possible to construct only 6 *CYP2C19* haplogenotypes (rs12248560/rs4244285). Nevertheless, Ionova et al. (30) detected 0.045‰ individuals from the population of 2.3 million participants to be carriers of *CYP2C19* ^*^1^*^17/^*^2^*^2, ^*^17^*^17/^*^2^*^2, or ^*^17^*^17/^*^1^*^2 haplogenotypes with unknown phenotype.

**Figure 1:**
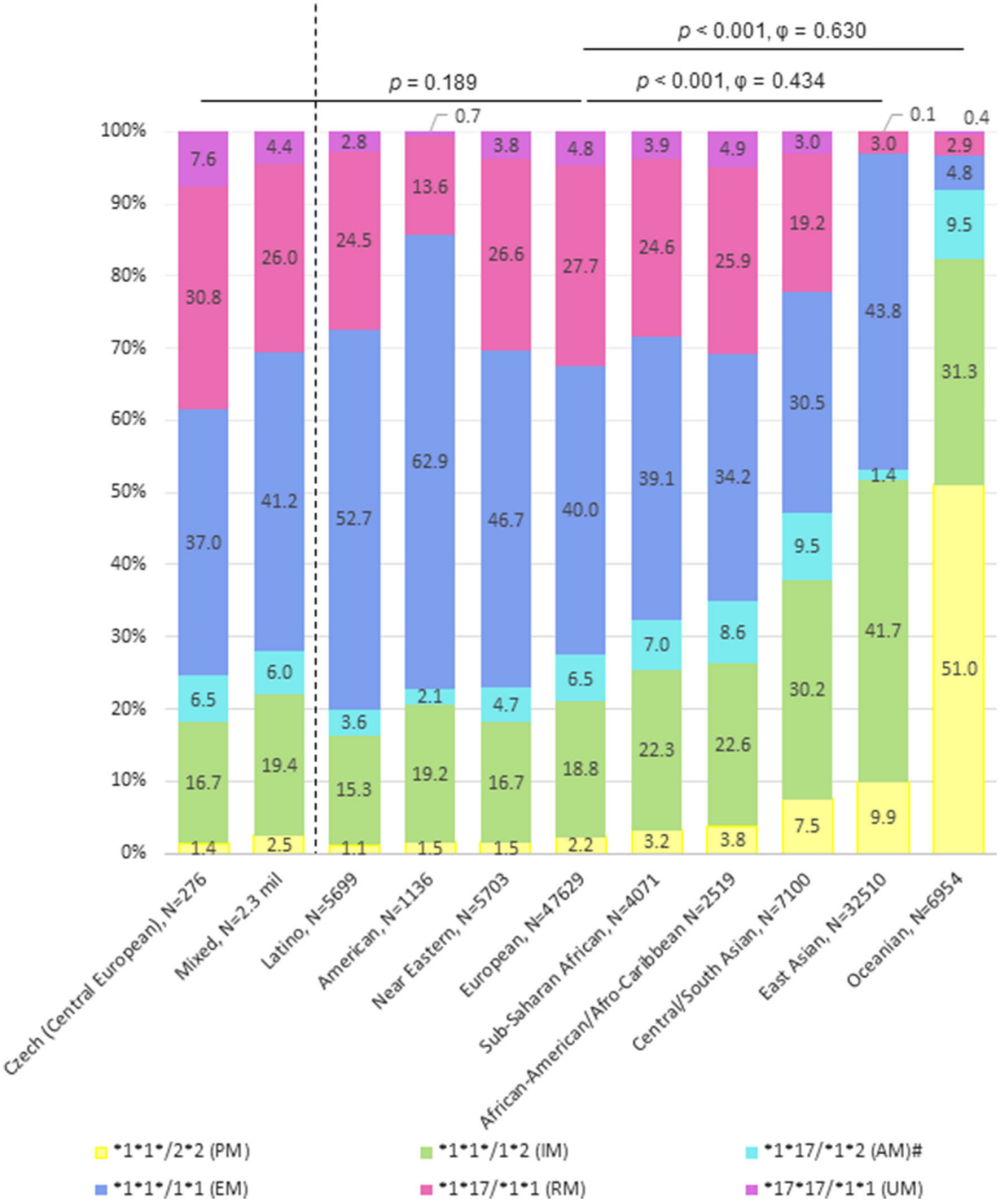
Distribution of the *CYP2C19* haplogenotype frequencies affecting proton pump inhibitors (PPI) metabolization in various populations. PM, poor metabolizer; IM, intermediate metabolizer; AM, ambivalent metabolizer; EM, extensive metabolizer; RM, rapid metabolizer; UM, ultrarapid metabolizer; CYP, cytochrome P450. The dotted line divides the figure into two populations in which haplogenotypes were determined experimentally (29, 30), and nine populations in which the haplogenotypes were calculated from a database containing haplotype (diplotype) frequencies estimated using the equation describing Hardy Weinberg equilibrium based on reported allele frequencies (22). Six *CYP2C19* haplotypes/predictive phenotypes results from combination of two *CYP2C19* polymorphisms (rs12248560 for detection of allele CYP2C19*17 / rs4244285 for detection of allele CYP2C19*2). ^#^*CYP2C19* *1*17/*2*2, *17*17/*2*2, and *17*17/*1*2 haplogenotypes with unknown phenotype are merged with AMs.

We calculated and compared *CYP2C19* haplogenotype frequencies in different populations using the PharmGKB database, see Figure 1. Results obtained experimentally in our previous study in Czech (Central European) adults with gastroesophageal reflux disease are in line with the *CYP2C19* haplogenotypes (rs12248560/rs4244285) calculated for the European population (*p* = 0.180). Only the East Asian (*p* < 0.001, ϕ = 0.434) and Oceanian population (*p* < 0.001, ϕ = 0.630) differed with medium and large effect, respectively, from the European population. While the representation of PMs ranges from 1.1 to 2.2% in Latino, American, Near Eastern, and European populations, it is approx. 9.9% in the East Asian and as much as 51.0% in the Oceanian population. On the other hand, the frequency of UM individuals in these two populations is very low (0.1 and 0.4%, respectively). The highest frequencies of the allele ^*^17 carriers (i.e., RMs and UMs) were calculated for African, European and Near-Eastern populations.

## Discussion

Most drugs prescribed for SUP fall within three principal groups: proton pump inhibitors (PPIs), histamine H_2_ receptor antagonists (H_2_RAs), and gastric mucosa protectants (GMPs). In a recent systematic review and meta-analysis comparing SUP agents with placebo and no-treatment arms in ICU patients, Wang et al. (41) evaluated (in a cohort of 39,569 critically ill patients from a total of 74 trials) the efficacy and safety of individual SUP agents. Results of this meta-analysis demonstrate that PPIs and H_2_RAs have the same effect on mortality compared to no prophylaxis. Both groups of SUP agents reduced clinically important GIB, with the PPIs being somewhat more effective than H_2_RAs. Liu et al. (42) also found in their non-ICU population that the best results were achieved with PPIs, which were superior to H_2_RAs as well as to GMPs in the GIB prevention. Moreover, in comparison with other agents, such as anticholinergics, synthetic prostaglandin analogs, or H_2_RAs, PPIs show good tolerance, safety, and a generally superior acid-suppressing activity. For these reasons, PPIs appear to be the SUP agent of choice unless contraindicated; still, it is necessary to mention that none of the prophylactic strategies can completely eliminate the risk of GIB.

In clinical practice, however, the general awareness of all PPI options and differences in their action and pharmacokinetics is relatively low. Typically, in practice, time-proven PPIs such as omeprazole or lansoprazole are prescribed (as they are the most widely studied and highlighted in current intensive care guidelines) and new developments are largely disregarded, although, in most countries, the range of PPIs offered by distributors is relatively wide (43–46).

PPIs in SUP must be chosen very carefully and personalized approach should be always considered. Most PPIs are degraded by the CYP2C19 enzyme, the genetic variability of which is quite common and bears a significant functional impact. For these reasons, the individual patients' classification according to their *CYP2C19* gene profile is a suitable tool for more personalized PPIs treatment. Dean and Kane summarized in their latest guidelines the dosing recommendations for omeprazole, taking into account the *CYP2C19* gene variability; nevertheless, there are no recommendations for specific agent selection in this indication that would consider the patient's individual genetic predispositions. Information for personalized PPIs treatment can be found in several databases; we assembled them and prepared a set of recommendations for personalized PPI-based stress ulcer prophylaxis taking into account the patient's CYP2C19 predictive phenotype, see also Supplementary Figure 1.

All PPIs have the same contraindications and precautions for their use. PPIs are contraindicated in case of hypersensitivity, which can be crossed among various PPIs. A higher risk of hypersensitive reactions could be expected when other drugs with the structure of substituted benzimidazoles (e.g., anthelmintics, H1 antihistamines) are co-administered. The immediate (urticarial/angioedema, anaphylaxis) and delayed hypersensitive reactions (Drug Reaction with Eosinophilia and Systemic Symptoms, Steven-Johnson syndrome/toxic epidermal necrolysis) are reported as severe adverse events associated with PPI therapy that can develop even after short-term use (47–53). Reports of more severe adverse effects including kidney disease, fractures, infections and vitamin deficiencies are very rare and are generally associated with long-term use (54, 55). Common adverse effects of PPI administration include headache, abdominal pain, constipation, diarrhea, flatulence, nausea/vomiting, and fundic gland polyps (benign). Findings from a meta-analysis also indicate a significant risk of incident *Clostridium difficile* infection among PPI users (56), although the causality is unclear. In the case of known hypersensitivity to PPIs or severe adverse effects of PPI therapy, therefore, other SUPs (H_2_RA, GMP) should be used.

Based on the information in the individual summaries of product characteristics, pantoprazole bears the lowest risk of common adverse effects (incidence ≥1/100 to < 1/10), followed by omeprazole and esomeprazole, lansoprazole, and rabeprazole with the highest number and variety of adverse drug reactions. Pantoprazole and omeprazole can also be considered preferred PPIs in terms of costs per daily dose and, with respect to safety profiles and clinical evidence, could be the drugs of choice among PPIs (57, 58).

Although PPIs are generally well-tolerated, the genetically determined risk of adverse effects resulting from a long-term therapy in patients with a decreased function of the CYP2C19 enzyme should not be neglected. While large epidemiological studies do not provide insight into the risk relative to *CYP2C19* genotype, given that there does appear to be a link between the dose and risk, it is plausible that these PPI-related adverse events are associated with *CYP2C19* PM/IM phenotype impeding the clearance of the drug from the organism. Two studies in children using lansoprazole reported an increased incidence of respiratory adverse events in PMs/IMs in comparison to EMs (38, 59).

CPIC guidelines recommend considering a 50% reduction in the daily dose of first-generation PPIs to minimize the risk of adverse effects for chronic therapy (>12 weeks) in PMs, IMs and AMs (20). However, the data for determining the time point for reduction of the daily dose are insufficient. It follows that rabeprazole, which is dominantly degraded non-enzymatically, seems to be the most reasonable choice for SUP in ICU patients with a genetic predisposition to decreased function of the CYP2C19 enzyme. Similarly, rabeprazole use may be also suggested as the first choice for patients using a drug with possible interaction via CYP3A4 and/or CYP2C19 enzymes and, generally, for SUP if the *CYP2C19* genotyping is not available (60, 61).

So far, there is a lack of information about the phenotype of the carriers of the combination of both *CYP*2*C*19^*^17 and *CYP*2*C*19^*^2 alleles. Although it appears that such a phenotype is likely similar to that of IM (37), it has not been proven yet and must be determined experimentally by the therapeutic drug monitoring method. Until such time, therapy independent of the CYP2C19 enzyme should be preferred (i.e., rabeprazole or a drug from other SUP groups). On the other hand, in UM patients, most PPIs are metabolized too fast and the effective concentration of the drug is, therefore, not maintained. In such individuals, only rabeprazole can be recommended in the standard dose while first-generation PPIs should be administrated at a daily dose increased by 100% (22). In our opinion, the use of rabeprazole appears to be an especially reasonable choice in *H. pylori*-positive ICU patients with UM predictive phenotype, thanks to its good *H. pylori* eradication rates.

While rabeprazole seems to be the most universal PPI in relation to a patient's *CYP2C19* gene profile, omeprazole is the most versatile PPI from the perspective of individualized therapy, being available in most countries in three different drug strengths and various formulations for oral and parenteral use. In addition, there are other PPIs, namely dexlansoprazole, tenatoprazole and ilaprazole, the information about their metabolism is, however, still limited. Given the similarity in metabolization between lansoprazole and dexlansoprazole, it is reasonable to extrapolate the recommendations from the first-generation PPIs (22). The advantage of tenatoprazole lies in its long half-life and longer duration of antisecretory action (62); however, data about the impact of *CYP2C19* polymorphism on its metabolization are largely unavailable. Pharmacokinetics and pharmacodynamics of ilaprazole, which is primarily metabolized by CYP3A4 and CYP3A5 (63), are not significantly influenced by the *CYP2C19* polymorphism (64).

Based on the genetic analysis of populations, the recommendation to determine *CYP2C19* haplogenotypes (rs12248560/rs4244285) before SUP using PPIs is expected to be most beneficial for populations with high representations of the *CYP*2*C*19^*^17 allele, i.e., Near-Eastern, African, and European populations with a 35.1–39.4% frequency, respectively, see also Figure 1. In these populations with a (relatively) high frequency of the ^*^17 allele, the rate PPI metabolization is highly variable and patient genotyping has the highest potential to affect the choice of PPI. This is, on the other hand, not so for (e.g.,) the East Asian and Oceanian populations, where only ~3% of patients are likely to have rapid or ultrarapid metabolization (22). Of course, it would be optimal to perform genotypization also in these populations; however, the cost-benefit ratio of genotypization in these populations would be very low as it would affect the treatment only in a tiny fraction of patients.

In conclusion, understanding pharmacokinetic differences and investigation of possible alternative metabolizing pathways can help better individualize SUP in critically ill patients. The increase in costs represented by the determination of both *CYP*2*C*19^*^17 and *CYP*2*C*19^*^2 polymorphisms before PPI administration compared to the potential benefits to the patients is relatively negligible. The suggested set of recommendations for personalized PPI-based stress ulcer prophylaxis taking into account the patient's CYP2C19 predictive phenotype may contribute to the implementation of the principles of personalized medicine into clinical practice.

## Data Availability Statement

The original contributions presented in the study are included in the article/Supplementary Material, further inquiries can be directed to the corresponding author/s.

## Author Contributions

PBL and BL designed this study. PBL, BL, and OZ wrote the manuscript. PBL, OZ, ZD, and NM prepared the figures. JJ, ZD, LB, and RK contributed to the critical review. MC analyzed the data. All authors read and approved the final manuscript.

## Funding

This study was supported by the Ministry of Health of the Czech Republic, grant No. NU20-03-00126. This research was supported by the Ministry of Health, Czech Republic – conceptual development of research organization (FNBr, 65269705). This publication has received funding from the European Union's Horizon 2020 Research and Innovation Programme under grant agreement No 857560. This publication reflects only the author's view and the European Commission is not responsible for any use that may be made of the information it contains. Authors also thank the Research Infrastructure RECETOX RI (No LM2018121) and project CETOCOEN EXCELLENCE (No CZ.02.1.01/0.0/0.0/17_043/0009632) financed by the Ministry of Education, Youth and Sports for supportive background.

## Conflict of Interest

The authors declare that the research was conducted in the absence of any commercial or financial relationships that could be construed as a potential conflict of interest.

## Publisher's Note

All claims expressed in this article are solely those of the authors and do not necessarily represent those of their affiliated organizations, or those of the publisher, the editors and the reviewers. Any product that may be evaluated in this article, or claim that may be made by its manufacturer, is not guaranteed or endorsed by the publisher.

## Abbreviations

ICU: intensive care unit
GIB: gastrointestinal bleeding
SUP: stress ulcer prophylaxis
PPI: proton pump inhibitor
FDA: Food and Drug Administration
CYP: cytochrome P450
CPIC: Clinical Pharmacogenetics Implementation Consortium
DPWG: The Dutch Pharmacogenetics Working Group
KNMP: Royal Dutch Pharmacists Association
SNP: single nucleotide polymorphism
UM: ultrarapid metabolizer
RM: rapid metabolizer
EM: extensive metabolizer
IM: intermediate metabolizer
PM: poor metabolizer
AM: ambivalent metabolizer
PCR: polymerase chain reaction
H_2_RA: histamine H_2_ receptor antagonist
GMP: gastric mucosa protectant
DRESS: Drug Reaction with Eosinophilia and Systemic Symptoms
SJS: Stevens-Johnson syndrome
TEN: Toxic Epidermal Necrolysis.
