# Supplementary material for: CYP2C19 Gene Profiling as a Tool for Personalized Stress Ulcer Prophylaxis With Proton Pump Inhibitors in Critically Ill Patients - Recommendations Proposal
Source: Front Med (Lausanne). 2022 Jul 11;9:854280. doi: 10.3389/fmed.2022.854280 (PMC9309431; doi:10.3389/fmed.2022.854280)
Supplement: Supplementary file 1 [file Table_1.docx]

**Supplementary Table 1.** Six CYP2C19 predictive phenotypes based on two *CYP2C19* polymorphisms (rs12248560 for detection of allele *CYP2C19**17 / rs4244285 for detection of allele *CYP2C19**2)

|  | **SNP in promotor**  **rs12248560** | **SNP in exon 5**  **rs4244285** |
| --- | --- | --- |
| **Allele (function)** | C = *1 (standard)  T = *17 (increased) | G = *1 (standard)  A = *2 (decreased) |
| **Haplotype** | CG = *1*1  CA = *1*2  TG = *17*1  TA = *17*2 | |
| **Genotype** | CC = *1*1  CT = *1*17  TT =*17*17 | GG =*1*1  GA = *1*2  AA = *2*2 |
| **Haplogenotype**  **(Predictive phenotype)** | CC/AA = *1*1/*2*2 (PM)  CC/GA = *1*1/*1*2 (IM)  CC/GG = *1*1/*1*1 (EM)  CT/GG = *1*17/*1*1 (RM)  CT/GA = *1*17/*1*2 (AM - unknown)  CT/AA = *1*17/*2*2 (unknown)  TT/GG = *17*17/*1*1 (UM)  TT/AG = *17*17/*1*2 (unknown)  TT/AA = *17*17/*2*2 (unknown) | |

PM, poor metabolizer; IM, intermediate metabolizer; AM, ambivalent metabolizer; EM, extensive metabolizer; RM, rapid metabolizer; UM, ultrarapid metabolizer; CYP, cytochrome P450

Haplotype (called diplotype in case of only two involved SNPs) is a combination of alleles inherited together from a single parent, present on the same DNA strain.

Haplogenotype represents a combination of selected genotypes, which can be associated with a specific phenotype.
