# Supplementary material for: CYP2C19 Gene Profiling as a Tool for Personalized Stress Ulcer Prophylaxis With Proton Pump Inhibitors in Critically Ill Patients - Recommendations Proposal
Source: Front Med (Lausanne). 2022 Jul 11;9:854280. doi: 10.3389/fmed.2022.854280 (PMC9309431; doi:10.3389/fmed.2022.854280)
Supplement: Supplementary file 2 [file Image_1.pdf]

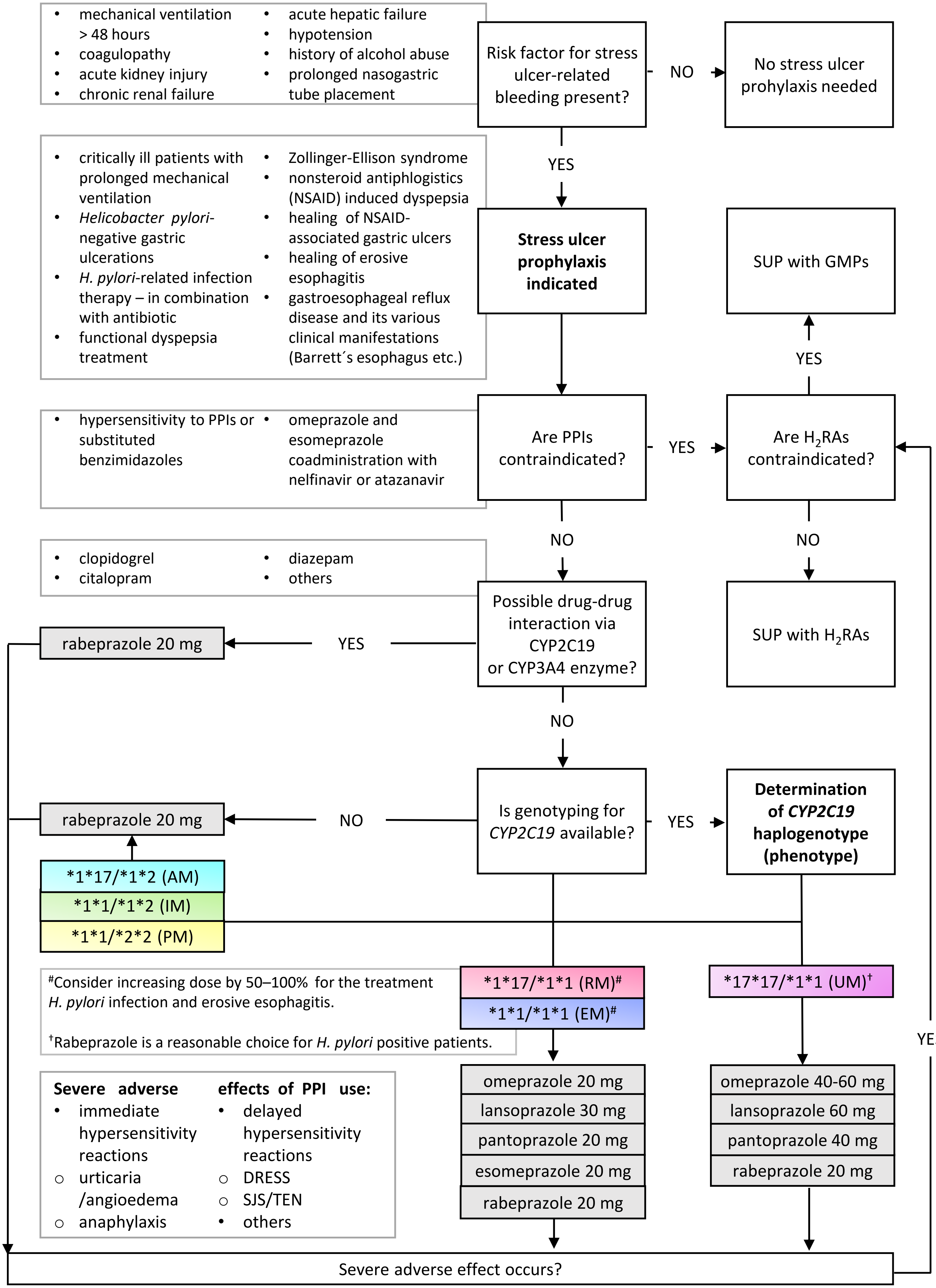

Supplementary Figure 1. The flowchart with proton pump inhibitors (PPIs) dosage recommendations for stress ulcer prophylaxis (SUP) in critically ill patients according to their *CYP2C19* gene profile

PM, poor metabolizer; IM, intermediate metabolizer; AM, ambivalent metabolizer; EM, extensive metabolizer; RM, rapid metabolizer; UM, ultrarapid metabolizer; CYP, cytochrome P450; GMP, gastric mucosa protectant; H_2RA, histamine H_2 receptor antagonist; DRESS, Drug Reaction with Eosinophilia and Systemic Symptoms; SJS, Stevens-Johnson Syndrome; TEN, Toxic Epidermal Necrolysis

Six *CYP2C19* haplotypes/predictive phenotypes result from the combination of two *CYP2C19* polymorphisms (rs12248560 for detection of allele *CYP2C19**17 / rs4244285 for detection of allele *CYP2C19**2)
